# Phagocytosis and activation of bone marrow‐derived macrophages by *Plasmodium falciparum* gametocytes

**DOI:** 10.1186/s12936-021-03589-2

**Published:** 2021-02-10

**Authors:** Yolanda Corbett, Silvia Parapini, Federica Perego, Valeria Messina, Serena Delbue, Paola Misiano, Mario Falchi, Francesco Silvestrini, Donatella Taramelli, Nicoletta Basilico, Sarah D’Alessandro

**Affiliations:** 1 Dipartimento di Scienze Farmacologiche e Biomolecolari, Università degli Studi di Milano, via Pascal 36, 20133 Milan, Italy; 2 Dipartimento di Scienze Biomediche per la Salute, Università degli Studi di Milano, Milan, Italy; 3 Dipartimento di Scienze Biomediche, Chirurgiche e Odontoiatriche, Università degli Studi di Milano, via Pascal 36, 20133 Milan, Italy; 4 Dipartimento di Malattie Infettive, Istituto Superiore di Sanità, Rome, Italy; 5 AIDS-Ricerca e sviluppo, Istituto Superiore di Sanità, Rome, Italy; 6 Centro Interuniversitario di Ricerca sulla Malaria-Italian Malaria Network, Milan, Italy; 7 Present Address: Dipartimento di Scienze Farmacologiche e Biomolecolari, Università degli Studi di Milano, via Pascal 36, 20133 Milan, Italy

**Keywords:** Malaria, *Plasmodium falciparum* gametocytes, Immortalized mouse C57Bl/6 bone marrow-derived macrophages, Phagocytosis, Nitric oxide, Tumour necrosis factor-alpha, Macrophage inflammatory protein 2

## Abstract

**Background:**

The innate immune response against various life cycle stages of the malaria parasite plays an important role in protection against the disease and regulation of its severity. Phagocytosis of asexual erythrocytic stages is well documented, but little and contrasting results are available about phagocytic clearance of sexual stages, the gametocytes, which are responsible for the transmission of the parasites from humans to mosquitoes. Similarly, activation of host macrophages by gametocytes has not yet been carefully addressed.

**Methods:**

Phagocytosis of early or late *Plasmodium falciparum* gametocytes was evaluated through methanol fixed cytospin preparations of immortalized mouse C57Bl/6 bone marrow-derived macrophages treated for 2 h with *P. falciparum* and stained with Giemsa, and it was confirmed through a standardized bioluminescent method using the transgenic *P. falciparum* 3D7elo1-pfs16-CBG99 strain. Activation was evaluated by measuring nitric oxide or cytokine levels in the supernatants of immortalized mouse C57Bl/6 bone marrow-derived macrophages treated with early or late gametocytes.

**Results:**

The results showed that murine bone marrow-derived macrophages can phagocytose both early and late gametocytes, but only the latter were able to induce the production of inflammatory mediators, specifically nitric oxide and the cytokines tumour necrosis factor and macrophage inflammatory protein 2.

**Conclusions:**

These results support the hypothesis that developing gametocytes interact in different ways with innate immune cells of the host. Moreover, the present study proposes that early and late gametocytes act differently as targets for innate immune responses.

## Background

Malaria is a vector-borne parasitic disease caused by the protozoan *Plasmodium* and transmitted by female *Anopheles* mosquitoes. In 2019, 229 million cases of malaria and 409,000 deaths have been reported, mostly due to *Plasmodium falciparum* and *Plasmodium vivax*, two of the five species which infect humans [1].

The life cycle of *Plasmodium* involves two hosts, the mosquito vector and a vertebrate. In humans, parasites develop in the liver without symptoms for the host, whereas the symptomatic phase starts with the asexual replication inside red blood cells. After rounds of asexual intraerythrocytic replication, a small proportion of parasites develop into sexual forms called gametocytes (GCT). Male and female GCT undergo five stages of differentiation, from I to V [2]. Stage V GCT circulate for several days until they are taken up by the vector, where the sporogonic sexual cycle occurs leading to the production of sporozoites, which can be injected into a new host. GCT have been found in the bone marrow (BM), spleen, gut, and brain of post-mortem samples from malaria patients, although the presence in BM is much higher and reported in different studies [3, 4]. GCT are also present in the BM of malaria patients [5, 6], including anaemic children [7].

Differently from *P. falciparum* asexual blood stages, which sequester in the deep vasculature via specific endothelial receptors [8], GCT lack specific adhesion molecules, do not adhere to the endothelium nor to primary erythroblasts, and have been found in the BM extravascular space [3, 5, 9–12]. The presence of *Plasmodium* GCT in the BM suggests that this microenvironment may sustain both infection and transmission [13–15]. However, at present, the exact molecular mechanism of GCT sequestration in the BM is not clear. The BM niche where GCT reside is characterized by the presence of many cell types, both haematopoietic and non-haematopoietic. Among these, macrophages play a crucial role in haematopoietic stem cells retention and survival and erythroid maturation [16]. One of the hypotheses concerning the presence of gametocytes in the BM is that they develop inside erythroblasts, thus affecting erythropoiesis [17]. In the BM of malaria patients, mature asexual parasites and haemozoin, the malarial pigment, are also found [18].

There is a gap of knowledge on the role of macrophages in the development and survival of GCT as well as on the effect of GCT on the activation and functions of BM macrophages.

Macrophages are the major effector of innate immunity. In malaria, splenic macrophages are responsible for the direct and antibody-dependent elimination of infected red blood cells (RBC) [19, 20]. Monocytes/macrophages from different organs are also activated by parasite antigens or products to release cytokines and chemokines and toxic mediators [21]. However, most of the data in the literature describe the response of macrophages to asexual blood-stage parasites. Contrasting data are available on the relationship between GCT and macrophages, on GCT phagocytosis, and macrophage activation by GCT [22–25].

The present work aimed to investigate some macrophage responses induced by GCT in BM, the place where GCT develop. In particular, the interactions between BM macrophages and early or late GCT were studied through the examination of phagocytosis of GCT and the induction of pro-inflammatory cytokines or nitric oxide (NO).

## Methods

### Reagents

Unless otherwise stated, reagents for cell and parasite culture were from EuroClone S.p.A (Pero, Italy), whereas all other reagents were from Sigma Aldrich S.r.l. (Milano, Italy).

### Cell culture

Immortalized mouse C57Bl/6 BM-derived macrophages (BMDM) from wild type lineage were kindly provided by Drs. Douglas Golenbock and Kate Fitzgerald, UMASS (MA, USA) [26, 27]. *Mycoplasma*-free BMDM were maintained in Dulbecco’s minimal essential medium (DMEM) complemented with 2 mM L-glutamine, 20 mM HEPES, and 10 % heat-inactivated FBS, as previously reported. The cultures were maintained at 37 °C in an atmosphere with 5 % CO_2_.

### Parasites culture

The transgenic *P. falciparum* 3D7elo1-pfs16-CBG99 strain expressing the *Pyrophorus plagiophthalamus* CBG99 luciferase under the gametocyte specific promoter pfs16 was used in all the experiments [28, 29]. The strain was kindly provided by Dr Pietro Alano at ISS (Rome, Italy). *Mycoplasma*-free *P. falciparum* parasites were maintained *in vitro* at 5 % haematocrit (human type 0-positive) RBC in RPMI-1640 supplemented with 10 % (v/v) naturally-clotted heat-inactivated (human type 0-positive) (Interstate Blood Bank, Inc. [USA]) serum, 0.37 mM hypoxanthine, 2 mM L-glutamine, and 25 mM HEPES. The cultures were maintained at 37 °C in a standard gas mixture consisting of 1 % O_2_, 5 % CO_2_, and 94 % N_2_ [30].

GCT were obtained as previously described [30]. Briefly, asexual parasites were diluted with fresh RBC and the medium was changed daily without further addition of RBC, to obtain a parasitaemia higher than 5 %. The cultures were treated with 50 mM N-acetylglucosamine (NAG) to clear residual asexual parasites and to obtain virtually pure GCT cultures. Early GCT (stage I–III GCT) were obtained after 4–5 days of NAG treatment, while late GCT (> 95 % stage V) after 12–14 days. The GCT stages were identified and counted through Giemsa-stained smears.

### GCT purification

GCT were purified by magnetic separation using MACS columns (Miltenyi®), based on the paramagnetic properties of haemozoin, which is present in GCT. Cultures at ~ 0.5 % haematocrit were placed onto an LS column to retain and remove the free haemozoin, as described earlier [31]. The LS column elution, which contains GCT, was transferred onto an LD column, where GCT remained attached. Finally, GCT were eluted from this column with fresh medium and then centrifuged. Both total RBC and GCT were counted at an optical microscope in a Neubauer cell counting chamber. The percentage of parasitaemia was confirmed in Giemsa-stained smears.

### Incubation of immortalized mouse C57Bl/6 bone marrow-derived macrophages with GCT

BMDM were plated in 96-well plates at a concentration of 10^5^ cells/well in 100 µl/well of cell medium and left adhere overnight. Experiments for luminescence reading were performed in 96-well white plates, whereas experiments for NO/cytokines/chemokines production in transparent plates. Only for evaluation of NO production, cells were pre-treated with interferon-gamma (IFN-γ) (100 U/ml) for 2 h. BMDM were stimulated with medium, late (> 90 % stage V) or early GCT (stage I–III) for 2 h in shaking conditions. In some experiments, cells were pre-treated for 1 h with 2 µM cytochalasin D to inhibit phagocytosis. GCT were added to cells in 100 µl/well volume in the GCT medium. The cell:GCT ratio was 1:1.5-2.

Uninfected RBC at the same haematocrit were used as controls in all the experiments. Lipopolysaccharide (LPS) (100 ng/ml) was used as a positive control for NO/cytokine/chemokines production.

### Luminescent determination of GCT

GCT and BMDM were co-incubated for different time points, the supernatants were removed and cells were washed with PBS. To completely remove not-phagocytized GCT, cells were treated with ice-cold distilled water for 20 s, a treatment which does not affect cell viability, but osmotically lyses RBC, as described by Schwarzer and colleagues [32]. BMDM were washed with PBS to remove remaining traces of haemoglobin and to restore cellular physiologic conditions. Next, 70 µl of PBS and 70 µl of D-luciferin (1 mM in citrate buffer 0.1 M, pH 5.5) were added to each well and incubated for 20–30 min in the dark. Absolute Luminescent Units (ALU) were read with a microplate reader Synergy4 (Biotek®), using an integration time of 500 ms.

### Microscopic observation

BMDM were seeded in 24-well plates (2.5 × 10^5^/well in 600 µl), incubated with GCT, and then detached by trypsinization. Cells were centrifuged and cytospin was performed to spot cells onto slides. Alternatively, cells were plated and treated with GCT in 16-well chamber slides previously treated with poly-l-lysine MW70000-150000 (0.1 mg/ml) to increase cell adhesion (Labtek®). Slides were fixed in absolute methanol and stained with a 0.4 % Giemsa solution (w/v) prepared in buffered methanol solution with stabilizers and diluted in a pH 6.5 buffer. Images were taken with an inverted microscope (Nikon Ti-S) at 1000× magnification (immersion oil 100x objective) and the digital camera Nikon Digital Sight.

For confocal microscopy, BMDM were plated in 8-well or 16-well chamber slides previously treated with poly-l-lysine (0.1 mg/ml) and left adhere overnight. GCT were pre-treated for 30 min with 3 µM of a fluorescent marker (CellTracker™RedCMTPX Dye, Molecular Probes), then washed and incubated with BMDM for 2 h in shaking conditions. At the end of incubation, cells were washed with PBS and fixed with paraformaldehyde (formalin solution 4 % [Sigma]) for 15 min. After further PBS washing, cell membranes were stained with 10 µg/ml WGA-FITC (Wheat germ agglutinin, DBA Italia). Smears were mounted using the Fluoroshield™ mounting medium containing DAPI for nuclei staining. Images were taken on an inverted microscope equipped with a confocal spectral imaging system (Olympus Fluoview 1000, Tokyo, Japan) at 600× magnification.

### Determination of nitric oxide production by immortalized mouse C57Bl/6 bone marrow-derived macrophages

Accumulation of nitrite in cell supernatants was evaluated by Griess assay [33, 34] as an indirect measure of NO production, as described in different models reported by Corbett and colleagues [31]. The Griess reagent is a 50 % v/v mixture of Reagent A (1 % w/v sulphanilamide) and Reagent B (0.1 % w/v naphthyl ethylenediamine dihydrochloride and 2.5 % w/v phosphoric acid). One hundred microlitres of Griess reagent were added to 100 µl of sample supernatants. After 10 min incubation at room temperature, the absorbance was read at 540 nm, using a Synergy 4 microplate reader (Biotek, GE). A standard curve of sodium nitrite (NaNO_2_) was used to extrapolate the nitrite levels of samples.

### Determination of cytokine/chemokine production by immortalized mouse C57Bl/6 bone marrow-derived macrophages

The production of tumour necrosis factor (TNF) and macrophage inflammatory protein 2 (MIP-2) was evaluated using commercially available ELISA kits (R&D DuoSet) following the manufacturer’s instructions.

### Statistical analysis

Data were analysed by one-way or two-way ANOVA followed by posthoc multiple comparisons tests (Dunnett’s, Tukey’s), using the software GraphPad Prism 6, as reported in figure legends.

## Results

### Evaluation of GCT phagocytosis using a luminescent‐based bioassay

**Fig. 1:**
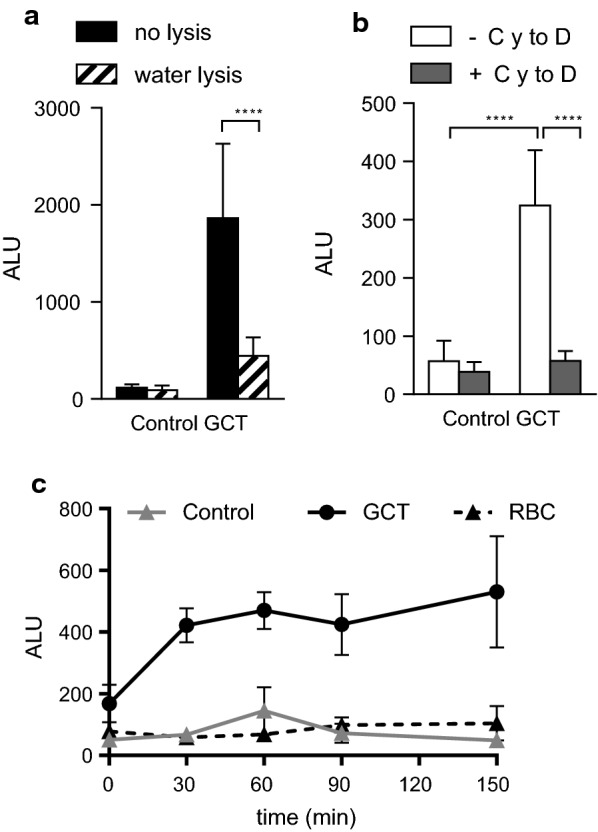
Set up of a luminescent assay to evaluate the phagocytosis of late GCT by immortalized mouse C57Bl/6 bone marrow-derived macrophages. **a** After 2 h incubation with GCT, cells were washed with water (water lysis, striped column) or PBS (no lysis, black column); **b** BMDM were pre-incubated in the presence (+ CytoD, grey column) or absence (-CytoD, white column) of Cytochalasin D (CytoD) 2 µM for 1 h before incubation with GCT. After 2 h incubation with GCT, the water lysis step was performed and luminescence was measured and expressed as Absolute Luminescent Units (ALU). **c** BMDM were incubated for different time points with GCT. After the water lysis step luminescence was measured and expressed as ALU. Data are the mean ± sd of 3 independent experiments in quadruplicate. Two-Way ANOVA, Tukey’s multiple comparisons test ****P < 0.0001

The bioassay to quantify GCT phagocytosis by BMDM was set up using luminescent late GCT (> 95 % stage V) from the transgenic *P. falciparum* 3D7elo1-pfs16-CBG99 strain cultured with BMDM in shaking conditions. The phagocytic activity of BMDM was confirmed by using latex beads. After 2 h of co-incubation, cells were washed to remove non-phagocytized parasites. To optimize the assay and eliminate the luminescent signal of non-phagocytized GCT, a lysis step with water was introduced before the addition of luciferin. The luminescent signal associated with macrophages was taken as a measure of GCT phagocytosis. As shown in Fig. 1a, a significant reduction in the luminescent signal was observed in the group exposed to lysis with water indicating that the lysis step is required to avoid unspecific signal and thus over-estimation of phagocytosis. The water lysis step has been performed in all the subsequent experiments.

To confirm that the luminescence signal was produced by the phagocytosed GCT, the second set of experiments was performed pre-incubating BMDM with Cytochalasin D, an inhibitor of actin polymerization and thus of cell phagocytosis [35]. As shown in Fig. 1b, in the presence of Cytochalasin D, the luminescent signal disappeared, indicating that the method is suitable to study phagocytosis.

Time course experiments were performed to assess gametocytes viability and luminescence after phagocytosis over time. Results indicated that luminescence of GCT inside BMDM increased from 0 to 30–60 min and remained stable up to 150 min, suggesting that GTC are still luminescent at 2 h after phagocytosis (Fig. 1c).

**Fig. 2:**
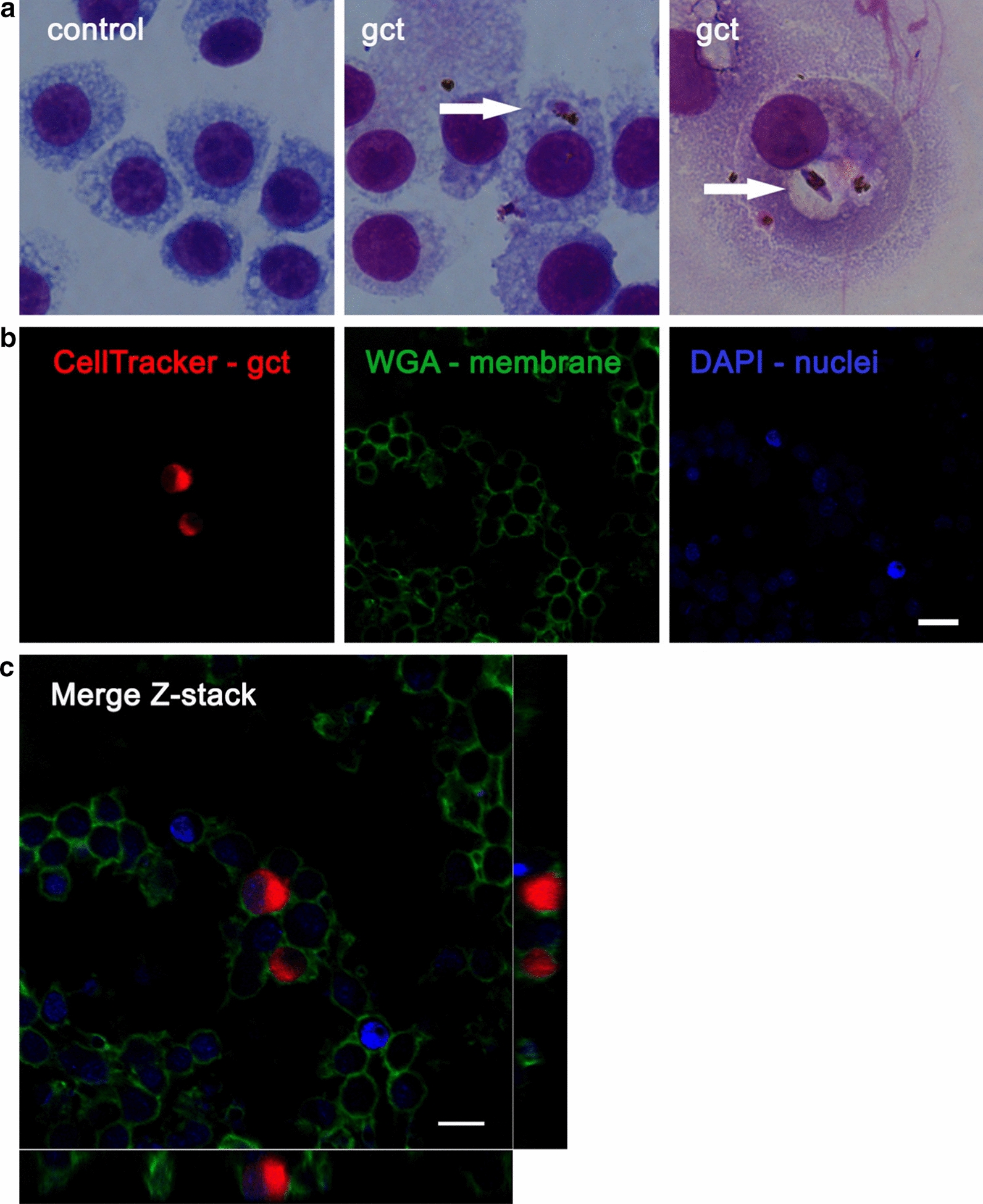
Microscopic observation of phagocytosis of late GCT by immortalized mouse C57Bl/6 bone marrow-derived macrophages. **a** BMDM were incubated with GCT for 2 h, detached from the plate, spotted on smears by cytospin centrifugation, fixed in absolute methanol, and stained with Giemsa. The arrows indicate GCT inside macrophages. Pictures, from one representative experiment out of three, were taken at ×1000 magnification. **b** GCT were incubated with 3 µM CellTracker™RedCMTPX Dye for 30 min to stain cytoplasm (red in the picture). BMDM were incubated for 2 h with GCT and fixed with formalin 4 %. BMDM membranes were stained with 10 µg/ml WGA-FITC (Wheat germ agglutinin, DBA Italia) (green in the picture). BMDM nuclei were stained by the DAPI present in the mounting medium (Fluoroshield) (blue in the picture). **c** Z-stack images were taken on an inverted microscope equipped with a confocal spectral imaging system (Olympus Fluoview 1000, Tokyo, Japan) 600× magnification. Scale bar, 10 µm

Phagocytosis of GCT was visualized and confirmed by different microscopic techniques. Figure 2a shows representative pictures of Giemsa-stained smears of BMDM containing late GCT in a vacuole in the cytoplasm after 2 h of co-culture. These data were confirmed by confocal microscopy analysis, where GCT, cell membranes, and nuclei were stained with different fluorescent markers to localize intracellular GCT. The presence of red GCT inside macrophages, stained green for membranes and blue for nuclei, was confirmed (Fig. 2b, c).

## Phagocytosis of early and late GCT

**Fig. 3:**
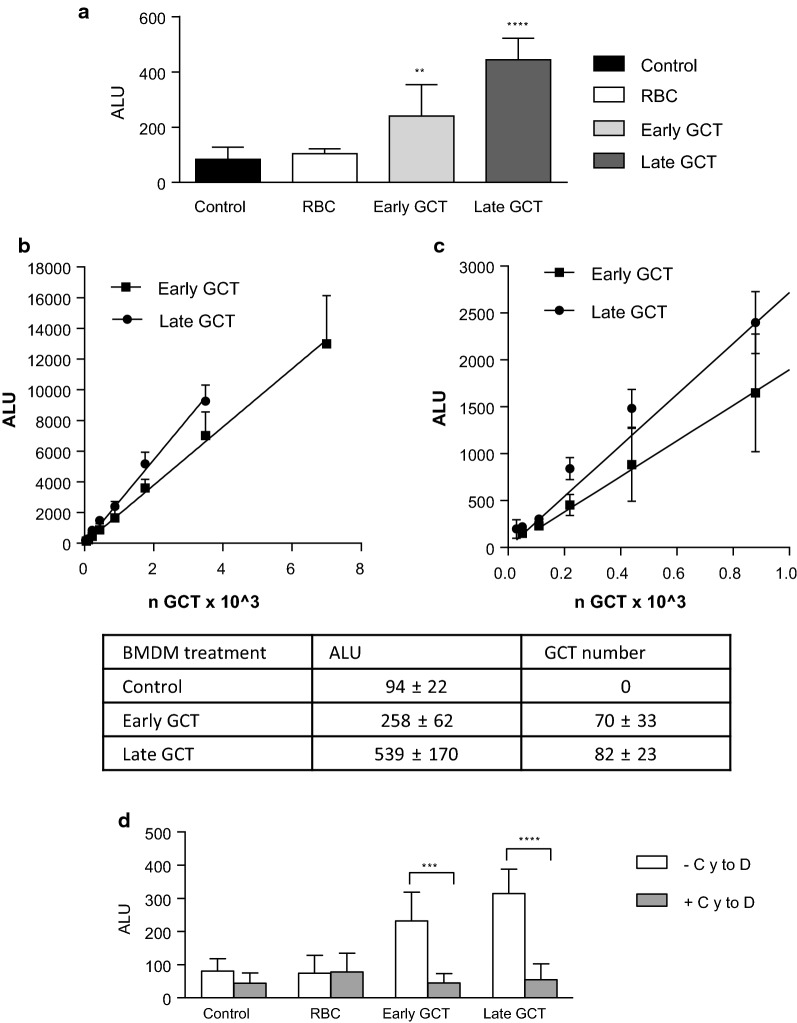
Immortalized mouse C57Bl/6 bone marrow-derived macrophages were treated with early or late GCT at the cell:GCT ratio of 1:1.5, for 2 h. **a** After washing and lysis, luminescence was read using a Biotek Synergy4 microplate reader. Results are expressed as arbitrary luminescence units (ALU). *p < 0.05; ****P < 0.0001 versus control, One-Way ANOVA, Dunnett’s multiple comparison test. **b** Linear correlation between the number of GCT and the relative luminescence in two different dose-response curves, one for late GCT, one for early GCT. **c** Magnification of the lower part of **b**. The table shows the luminescence emitted (ALU) by the medium alone, or by early or late GCT (BMDM treatment), and the numbers of GCT phagocytized by BMDM extrapolated from the dose-response curve shown in **c** (GCT number). **d** BMDM were pre-incubated in the presence (+ CytoD, grey column) or absence (−CytoD, white column) of Cytochalasin D (CytoD) 2 µM for 1 h before incubation with GCT. After 2 h incubation with GCT, the water lysis step was performed and luminescence was measured and expressed as Absolute Luminescent Units (ALU). ***p < 0.001; ****P < 0.0001 versus control, Two-Way ANOVA, Tuckey’s multiple comparison test. Data are the mean ± sd of 3 independent experiments in triplicate

The luciferase assay was utilized to evaluate the phagocytosis of late (> 90 % stage V) *versus* early (stage I–III) GCT. BMDM were incubated with late or early GCT for 2 h, at the cell:GCT ratio of 1:1.5. The luminescent signal was proportional to the number of GCT used in the assay. However, the luminescence was higher in the groups treated with late GCT compared to early GCT (Fig. 3a), suggesting greater phagocytosis of late GCT than early GCT.

To confirm the previous observations and be able to quantify the number of phagocytized GCT, the luciferase activity of serially diluted early or late GCT was evaluated. This was particularly crucial since it has been reported that the luciferase accumulates in 3D7elo1-pfs16-CBG99 GCT during their development, and thus the luminescent signal is higher in late versus early GCT [29].

As reported in Fig. 3b, a linear correlation between the number of GCT and the mean ALU values was observed. Late GCT mean ALU values were approximately 20–40 % higher than that of early GCT. The difference between early and late GCT and the linearity of the luciferase activity was maintained also at low GCT number and low ALU, as shown in Fig. 3c. These curves were used to extrapolate the number of GCT phagocytized by BMDM. The number of early and late GCT phagocytized by BMDM was 70 ± 33 and 82 ± 23, respectively. It appears that, when corrected for the luciferase activity of each GCT population, the extent of phagocytosis of early vs. late GCT was not significantly different.

The same experiments were performed pre-incubating BMDM with Cytochalasin D and, as expected, in the presence of Cytochalasin D the luminescent signal from BMDM incubated with gametocytes was significantly reduced and was comparable to controls (Fig. 3d).

### Production of nitric oxide and cytokines by immortalized mouse C57Bl/6 bone marrow-derived macrophages treated with GCT

**Fig. 4:**
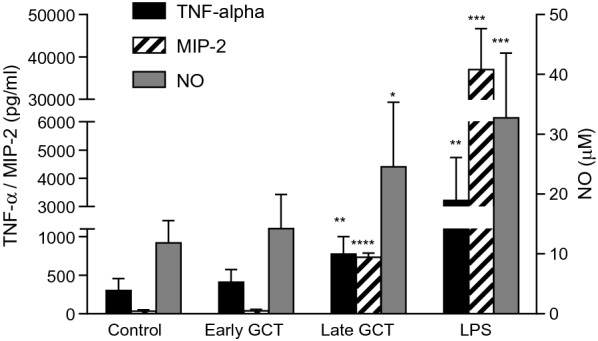
Immortalized mouse C57Bl/6 bone marrow-derived macrophages were treated with early or late GCT at the cell:GCT ratio of 1:2, for 24 h. The production of TNF and MIP-2 was evaluated in the supernatants by ELISA. Nitric oxide production was evaluated by measuring nitrite in cell supernatants by Griess assay. LPS (100 ng/ml) was used as a positive control. Data are the mean ± SD of 3 independent experiments in triplicate. *p < 0.05; **p < 0.01; ***p < 0.001; ****P < 0.0001 versus control, Two-Way ANOVA, Dunnett’s multiple comparison test

The ability of GCT to activate BMDM was evaluated by measuring the production of inflammatory mediators in the culture supernatants after 24 h. As shown in Fig. 4, late GCT induced a 2-fold increase in the production of both TNF and NO, compared to unstimulated controls. The CXC chemokine MIP-2 was also strongly induced. On the contrary, the stimulation of BMDM by early GCT was not significantly different from untreated controls. LPS (100 ng/ml) was used as a positive stimulus.

**Fig. 5:**
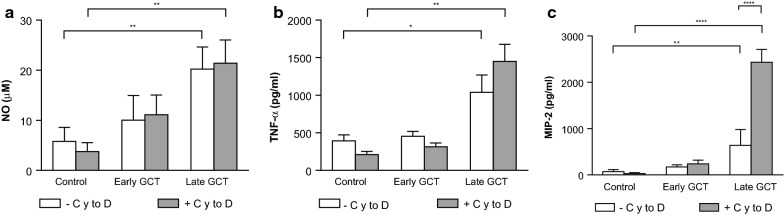
Immortalized mouse C57Bl/6 bone marrow-derived macrophages were pre-incubated in the presence (+ CytoD, grey column) or absence (-CytoD, white column) of Cytochalasin D (CytoD) 2 µM for 1 h before incubation with early or late GCT at the cell:GCT ratio of 1:2, for 24 h. The production of NO was evaluated by measuring nitrite in cell supernatants by Griess assay. The production of TNF and MIP-2 was evaluated in the supernatants by ELISA. Data are the mean ± sd of 3 independent experiments in triplicate. *p < 0.05; **p < 0.01; ***p < 0.001; ****P < 0.0001 *versus* control, Two-Way ANOVA, Tukey’s multiple comparison test

To address whether NO and cytokine production was due to phagocytosis or to contact with gametocytes, experiments were repeated in the presence of cytochalasin D. Results show that cytochalasin D did not modify the production of NO and increased the production of both TNF and MIP-2 induced by late GCT (Fig. 5) proving that phagocytosis was not involved in cytokines and NO induction. On the contrary, the introduction of a water lysis step abrogated the production of NO and cytokines induced by GCT indicating that NO and cytokines production was due to the contact of GCT and cells and not to the phagocytized parasites.

## Discussion

Gametocytes (GCT) have a key role in malaria, being the sexual blood stage of the parasite responsible for transmission. There is controversial information in the literature about the human immune responses elicited by GCT. The present study demonstrated that BMDM phagocytized both early and late *P. falciparum* GCT, but only late GCT induced the production of inflammatory mediators.

The phagocytosis of GCT by macrophages was observed by light microscopy on Giemsa-stained cytospin preparations and confirmed by confocal analysis, using different fluorescent probes to label and identify BMDM or GCT structures. The latter technique allowed the visualization of cells in 3 dimensions, which unequivocally determined the presence of GCT inside the cells. Moreover, the present work describes a simple bioluminescent method able to measure the presence of GCT inside BMDM. The transgenic 3D7elo1-pfs16-CBG99 *P. falciparum* strain expresses the potent luciferase CBG99 under the control of the GCT specific promoter Pfs16 and produces luminescence only in the GCT stage after the addition of luciferin [28]. The luminescence associated with BMDM after treatment with GCT was used as a measure of phagocytosis. To increase the sensitivity and specificity of the assay, the cells were carefully washed after phagocytosis of GCT, and lysis of non-phagocytized GCT was performed, preserving BMDM viability. Using an inhibitor of phagocytosis [35], the luminescent signal disappeared, confirming the reliability of the method. Data reporting phagocytosis of *P. falciparum* GCT in the literature are few and controversial, probably due to differences in the cellular models, isolation protocols, and GCT stage used to perform the experiments. Late GCT are not phagocytized by human differentiated THP-1 monocytes [23], whereas they are phagocytized by peripheral blood mononuclear cells, even if less efficiently compared to asexual stages or gametes [24]. Stage I-II GCT, but not stage IV GCT, are phagocytized by macrophages derived by human blood or murine peritoneal cavity with a CD36- dependent mechanism [22]. Up to now, phagocytosis of GCT by macrophages has been demonstrated mostly by using Giemsa staining and optical microscopy, a time-consuming method, highly dependent on operator skills. Moreover, optical microscopy does not allow discriminating precisely between parasites which are inside cells from parasites which are attached on the surface. All these limitations were overcome by using confocal microscopy and by the luminescent method proposed in this work.

The luminescent method is simple and reliable and was applied to compare the extent of phagocytosis of early *versus* late GCT. In this respect, the luminescence emitted by different numbers of early *versus* late GCT confirmed that the luminescent signal was higher in late compared to early GCT, as previously reported [29], but the extent of phagocytosis was similar. However, the efficiency of phagocytosis of both late and early stages was low, in line with the results of Healer and colleagues [24], which showed that phagocytosis of GCT was limited when compared to that of asexual parasites or gametes. The method can be easily applied to other *in vitro* models using transgenic luminescent microorganisms.

The observation here that GCT were phagocytized by BMDM reaffirms that innate immunity plays an important role in malaria. Several studies show that whole asexual *P. falciparum*-infected erythrocytes or their parasite products induce macrophages activation [21, 26, 36]. In particular, the malaria pigment, haemozoin, which is also present in GCT, is a potent pro-inflammatory stimulus for macrophages, leading to the activation of the toll-like receptors, inflammasome complex, and NOD2 pathway [26, 36, 37].

Anyhow, immune reactions may also involve sexual stages, affecting malaria transmission [25, 38–41]. Although the involvement of specific immunity mediated by antibodies against GCT and gametes antigens [42] was described [43], the role of innate immunity has not been well examined so far. Data from the literature show that there is a detrimental activity of cytokines or NO on GCT infectivity [38, 44].

The data presented here showed that late but not early GCT induced TNF, a pleiotropic cytokine with a range of activities [45, 46], including endothelial activation leading to the sequestration of asexual parasitized RBC in cerebral vessels during the severe forms of malaria [47, 48]. As stated before, several data from the literature reveal the absence of specific adhesion molecules on GCT. Rogers and colleagues showed that TNF increases the adhesion of early GCT to the endothelium of the BM [11]. Induction of TNF by late GCT described in the present manuscript suggested the potential role of this cytokine in the homing of early GCT to the BM, where they develop into late stages [3, 9–11].

In the same manner, only late GCT induced macrophage inflammatory protein MIP-2, a major CXC chemokine (CXCL2) involved in the recruitment of polymorphonuclear neutrophils to the sites of inflammation (reviewed in [49]). Previous studies from the authors of the present work showed that MIP-2 is upregulated in BMDM stimulated by malaria pigment [37]. This is the first evidence of the involvement of MIP-2 in GCT-macrophage interaction in BM. Matzer et al. [50] showed that MIP-2 is constitutively expressed in BM, but not in other organs like the spleen, lung, or liver. Interestingly, MIP-2 has a role in leukocyte adhesion in the BM [51]. Thus, MIP-2 induction by late GCT is worth further investigation.

Again, only late GCT induced NO production from BMDM. NO is a potent vasodilator and cellular mediator involved in many biologic functions, which participates in the pathogenesis of severe malaria and in particular of cerebral malaria [52]. NO is important not only in the human host but also in the vector since it contributes to the immune responses of mosquitoes against *Plasmodium* infection [53]. Moreover, NO increases parasite deformability, thus inducing the stage V GCT release from the BM niche towards circulation [54]. The results presented here sustain this hypothesis.

Even in the absence of GCT internalization, BMDM produced inflammatory mediators in response to GCT. Indeed, when cytochalasin D was used to prevent GCT phagocytosis, the production of NO was comparable in the presence and absence of cytochalasin D. The production of TNF and MIP-2 induced by GCT was even increased in the presence of cytochalasin D indicating that phagocytosis was not needed for BMDM stimulation. An increase of cytokines production by cells treated with cytochalasin D have been already described although the effect of cytochalasin D can be both stimulating and inhibiting depending on macrophages origin, type of pathogen and experimental model [55–57].

## Conclusions

The present work showed that early and late GCT are phagocytized by immortalized macrophages from BM cells but only late GCT induce the production of inflammatory mediators (i.e. TNF, MIP-2, or NO), regardless of phagocytosis. Thus, the data available in the literature, already discussed above, and the results presented here, suggest that innate immune responses elicited by late GCT, at least in BM cells, may have a dual role. On one side, they may establish a hostile milieu, inducing late GCT to escape from the bone marrow towards circulation. On the other side, they may support the retention and thus the development of early GCT in the bone marrow.

## Data Availability

Not applicable.

## Abbreviations

ALU: Absolute luminescent units
DMEM: Dulbecco’s minimal essential medium
early GCT: Stage I–III GCT
GCT: Gametocytes
BMDM: Immortalized mouse C57Bl/6 bone marrow (BM)-derived macrophages
IFN-γ: Interferon-gamma
late GCT: Stage V
LPS: Lipopolysaccharide
MIP-2: Macrophage inflammatory protein 2
NAG: *N*-Acetylglucosamine
NO: Nitric oxide
RBC: Red blood cells
TNF: Tumour necrosis factor
WGA: Wheat germ agglutinin
